# MEASUREMENTS OF SKIN CAROTENOID STATUS BY VEGGIE METER® IN URBAN ADULTS

**DOI:** 10.1093/geroni/igad104.2462

**Published:** 2023-12-21

**Authors:** Amy Schweitzer, Ricardo Brown, Lillie Monroe-Lord

**Affiliations:** University of the District of Columbia, Washington, District of Columbia, United States; UDC, Washington, District of Columbia, United States; University of the Dustrict of Columbia, Washington, District of Columbia, United States

## Abstract

Recent advances in technology related to objective measures of dietary intake have led to tools such as the Veggie Meter®. The Veggie Meter uses pressure-mediated reflection spectroscopy to determine skin carotenoid status as an objective proxy of fruit and vegetable intake. This project is part of a larger study to determine seasonal changes in skin carotenoids of residents in an urban setting. The purpose of this project is to report baseline measures of skin carotenoids in a subset of older urban adults. Adults frequenting community events (senior center, food pantries, health fairs) consented to triplicate Veggie Meter readings. Adults (n=54) ages >60 years old who provided consent completed a survey and a baseline Veggie Meter reading. Participants were 68% female with a mean ± standard deviation age of 68 ± 7.2 years. Mean BMI was 29.9 ± 7.47 kg/m² . Most were non-smokers (75%). Participants identified as African American (78%), Caucasian (7%), two or more races (7%), or other race (6%). Mean Veggie Meter score was 275.6 ± 98.88. Smokers had a significantly lower Veggie Meter® score (216±73.4) than non-smokers (295 ± 98.7) p=0.006. Conclusion: For this sample of older adults in the DC area, differences in skin carotenoid status was seen by smoking status. Additional data is pending for Winter, Spring and Summer readings to determine whether seasonal variations exist in skin carotenoid status as measured by the Veggie Meter.

